# Yield-Point Phenomenon and Plastic Bands in Ferrite–Pearlite Steels

**DOI:** 10.3390/ma16010195

**Published:** 2022-12-26

**Authors:** Hai Qiu, Rintaro Ueji, Tadanobu Inoue

**Affiliations:** Center for Structural Materials, National Institute for Materials Science, 1-2-1 Sengen, Tsukuba 305-0047, Ibaraki, Japan

**Keywords:** ferrite, pearlite, yield point, discontinuous yielding, plastic deformation, digital image correlation

## Abstract

Lüders deformation is one type of discontinuous yielding in ferrite–pearlite steel. The yield-point phenomenon and localized plastic bands are two features of the Lüders phenomenon. It is believed that the yield-point phenomenon is related to the formation of plastic bands, but the correlation between them is unclear. In this study, this correlation was investigated by examining the global and local deformation behaviors in the tension processes of four ferrite–pearlite steels (carbon content, 0.05–0.3%; pearlite fraction, 1.2–32%) via an extensometer and digital image correlation (DIC) technique. The main obtained results are as follows: (1) the degree of yield drop decreased with an increase in the pearlite fraction (the magnitude of the yield stress drop was 8.6–0 MPa), and (2) a plastic band was formed at a certain stress level smaller than the upper yield stress; when the stress level was larger than 92% of the upper yield stress, the upper yield point disappeared.

## 1. Introduction

Plastic instability occurring in the elastic-to-plastic transition region is usually called the Lüders phenomenon. The Lüders phenomenon has two typical appearances: (1) on the stress–strain curve, yield points and a yield plateau are present; and (2) on the surface of the bulk material, inhomogeneous deformation extends across the entire bulk material in the form of one single plastic band or multiple plastic bands. This Lüders phenomenon induces undesired stretcher-strain marks on the surface of sheets during metal-forming processes, which lowers the surface quality of the sheet products [1,2]. Therefore, investigating the mechanism of the Lüders phenomenon is important from an engineering viewpoint. 

The Lüders phenomenon was initially reported one hundred years ago, and the two aforementioned features were extensively studied fifty years ago. The conventional understandings of them are briefly summarized as follows: (1) Interstitial atoms such as C or N interact with dislocations, forming a “Cottrell atmosphere” around these atoms. Cottrell and Bilby regarded the yield point to be caused by the unpinning of dislocations from the atmosphere [3]. Hahn believed that rapid dislocation multiplication also can result in a yield point [4]. (2) The formation of the localized plastic band is related to the stress–strain curve. The upper yield stress is regarded as being the stress for the unlocking, creation, or rapid multiplication of mobile dislocations [5]. When the macroscopic applied stress reaches the upper yield stress, a plastic band begins to form in the material, i.e., the upper yield point is the beginning point of plastic band formation [5,6]. The lower yield stress is the stress that drives plastic band propagation toward the still unyielded region of the material [6]. 

Over the last two decades, research has been performed on the following aspects:

1. Studies on the yield-point phenomenon:
  (1) The micromechanism of the yield point.
    Shen et al. [7], Akama [8], and Tsuchiyama [9] investigated the yield-point phenomenon in low-carbon steels, and they found that carbon segregation [7] and grain boundary strengthening [8] significantly affect the occurrence of the yield point, and that the yield point is a dislocation emission phenomenon caused by dislocation sources that exist at the grain boundaries [9].
  (2) The development of yield-point models.
    Models have been proposed to describe the yield-point phenomenon based on the dislocation theory [10,11,12].
  (3) Effects of the second or third phases on the yield point in multiple phases.
    Commercial steels are generally composed of multiple phases. The effects of the second phase or third phase on the yield point have been investigated, such as cementite particles in ferrite–cementite steel [13,14], martensite islands in ferrite–martensite steel [13], and cementite in pearlite steel [15].
  (4) Influence of the yield point on the material’s behavior.
    Žerovnik et al. [16] investigated the effect of the yield-point phenomenon on the evolution of the cyclic plasticity of a low-alloy steel.
  (5) Elimination of the yield point.
    Metallurgical methods, such as recrystallization [17] or temper rolling [18], are useful for eliminating the yield point.
2. Studies on the plastic band:
  (1) The development of a micromechanical model of plastic bands.
    Schwab and Ruff [19] proposed a micromechanical model that revealed the relationship between the formation of plastic band and the upper and lower yield point.
  (2) In situ observations of moving plastic bands.
    The formation and propagation of plastic bands were observed in situ via a digital image correlation (DIC) technique [20,21,22]. It was found that plastic bands have been formed far ahead of the upper yield point [20,21].

The aforementioned recent research indicates that the conventional explanation of the yield point phenomenon, which is based on the dislocation theory, is rational. However, the in situ observation of plastic bands via the DIC technique shows that a plastic band can be formed at a stress level much smaller than the upper yield stress, which is inconsistent with the conventional understanding of the correlation between the band formation and the upper yield point (i.e., the upper yield point is the beginning point of band formation). This inconsistency leads to a question: what is the relationship between the yield point and the formation of plastic bands or, in other words, what is the correlation between the appearance (or disappearance) of the yield point and the formation of plastic bands? This problem is the focus of the present study. 

Ferrite–pearlite steel is a widely used structural steel, and the Lüders phenomenon is one of its popular deformation behaviors. In this study, we investigated the correlation between the yield point and the band formation in the ferrite–pearlite steel through the following methods: (1) controlling the yield-point phenomenon by varying the pearlite fraction, (2) observing in situ the deformation behavior via the DIC technique and an extensometer, and (3) revealing the critical conditions for the appearance (or disappearance) of the yield point. 

## 2. Materials, Tension Test Conditions, and Deformation Evaluation Methods

### 2.1. Materials

To obtain different volume fractions of pearlite, four steels were used in the present study. Their chemical compositions are given in Table 1. According to the carbon content, they are named 0.05C steel, 0.10C steel, 0.16C steel, and 0.3C steel. As few as possible additional elements were added to the 0.05C steel to produce pure ferrite steel. The conventional chemical composition of commercial steel sheets was used in the 0.10C, 0.16C, and 0.3C steels. Samples that were 120 mm long, 50 mm wide, and 1.2 mm thick were prepared from the four steels, and they were heat treated with the conditions shown in Table 1. The 0.05C steel, 0.10C steel, and 0.16C steel underwent the same heat-treatment procedure: samples were heated in a furnace at 900 ℃ for 5 min, and then were cooled down to room temperature in the furnace. The heating temperature was the only difference in the heat-treatment conditions between the 0.3C steel and the other three steels. The heating temperature for the 0.3C steel was 850 ℃ instead of 900 ℃. The microstructures of the four steels were examined with optical microscopy. Due to the difference in the carbon content, the pearlite contents of the four steels are different. 

### 2.2. Tension Test Conditions 

**Figure 1:**
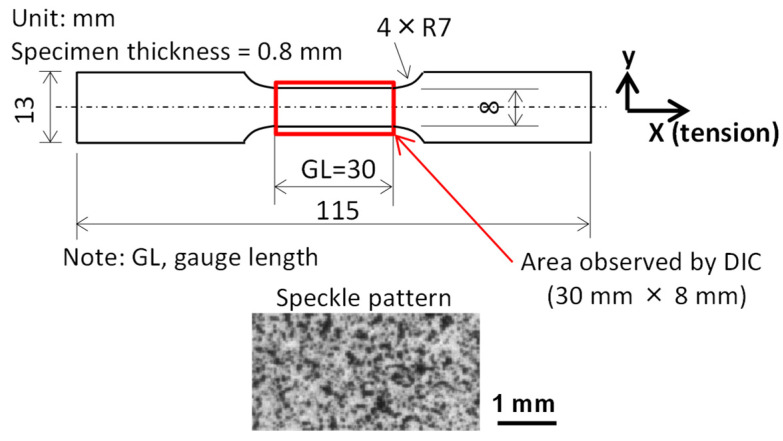
The size of the dog-bone-type specimen and the speckle patterns on the front surface of the specimen.

Dog-bone-type tensile specimens were machined from the heat-treated samples. The specimen size is shown in Figure 1. Before a tensile test, as shown in Figure 1, the front surface of the specimen was sprayed with white and black paint to make speckles for the DIC analysis, and an extensometer (type, EDP-10BLS-30; capacity, 10 mm; and gauge length, 30 mm; made by Tokyo Sokki Kenkyujo Co., Ltd., Japan) with a gauge length of 30 mm was attached to the back surface of the specimen. Tensile tests were carried out at room temperature and at a crosshead speed of 0.01 mm/s. The deformation process of the front surface over the whole parallel part of the specimen (30 mm × 8 mm) was recorded successively with a digital camera at a constant time interval of 500 ms. The direct outputs of the tension tests were as follows: (1) a global stress–strain curve; and (2) successive digital images describing the deformation process of the front surface of the tension specimens.

### 2.3. Deformation Evaluation Methods

The deformation of the bulk material was evaluated via the global strain and local strain. The global strain represented the global deformation behavior of the bulk material, and it was obtained with the extensometer as the average strain over the gauge length. The local strain showed the local deformation behavior, and it was used to identify the plastic strain concentration and plastic bands that occurred in the tension process. The local strain was derived from the DIC analysis, in which the obtained digital images were processed using VIC-2D software (produced by Correlated Solution, Inc., USA) with DIC parameters of a subset size of 9 pixels × 9 pixels (248 μm × 248 μm) and a step of 5 pixels (138 μm). 

## 3. Results and Discussion

### 3.1. Microstructure

**Figure 2:**
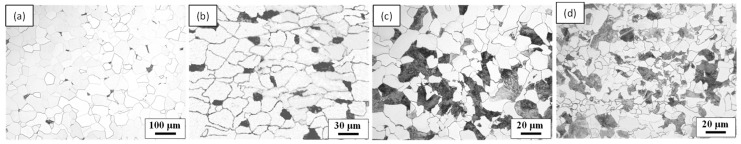
Optical microstructure of (**a**) 0.05C steel, (**b**) 0.10C steel, (**c**) 0.16C steel, and (**d**) 0.3C steel. Gray grains—ferrite; black grains—pearlite.

Figure 2 shows the optical microstructure of the (a) 0.05C steel, (b) 0.10C steel, (c) 0.16C steel, and (d) 0.3C steel. The three steels are composed of ferrite and pearlite. The ferrite grains are polycrystalline and equiaxial. The pearlite colonies in the 0.05C steel are isolated and dispersed in the ferrite matrix. As the volume fraction of pearlite increases, the tendency for the pearlite colonies to contact each other increases. Connected pearlite colonies are mainly seen in the optical microstructure of the 0.16C steel and 0.3C steel. The ferrite grain size and the volume fractions of cementite, ferrite, and pearlite are summarized in Table 2. The average ferrite grain sizes of the four steels are different: the 0.05C steel, 0.10C steel, 0.16C steel, and 0.3C steel are 30 μm, 18 μm, 9 μm, and 4 μm, respectively. The volume fraction of cementite and pearlite increases with the carbon content. The volume fractions of pearlite are 1.2%, 8%, 17%, and 32% for the 0.05C steel, 0.10C steel, 0.16C steel, and 0.3C steel, respectively; the corresponding volume fractions of cementite (f_ce_) in pearlite are 0.8%, 1.5%, 2.4%, and 4.6%, respectively. It is noted that the volume fraction of cementite was calculated by the formula f_ce_ = 1.946C/(12.72 + 0.046C), where C (carbon content) is in wt% [23]. 

Mizuno et al. [24] investigated the microstructures of ferrite–pearlite steels whose carbon contents vary from 0.001% to 0.611%. They found that a carbon content of 0.35% is a critical value that divides the carbon range into two regions: (1) below this value, the gap between the adjacent cementite layers in the pearlite colonies is 0.13–0.19 μm, and the volume fraction of pearlite linearly increases with the carbon content; and (2) above this value, the gap between the adjacent cementite layers in the pearlite colonies is 0.21–0.28 μm, and the volume fraction of pearlite nonlinearly increases with the carbon content. The volume fraction of pearlite in Table 2 agrees with their experimental data. The carbon content of the four steels used in the present study is below 0.35%; thus, the steels belong to the first group in Ref. [24]. According to these results, it is rational to neglect the difference between the four steels used in the gap between the adjacent cementite layers, as the volume fraction of pearlite is the main factor controlling the yielding behavior. 

### 3.2. Yield-Point Phenomenon

**Figure 3:**
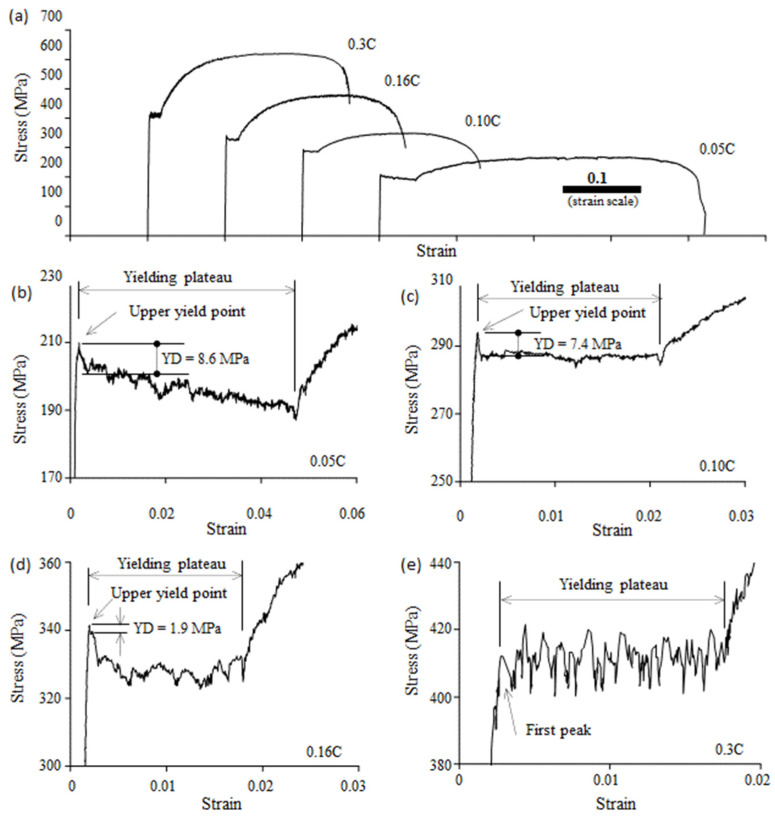
(**a**) Stress–strain curves of 0.05C, 0.10C, 0.16C, and 0.3C steels. Enlarged stress–strain curves around the yielding plateau of (**b**) 0.05C steel, (**c**) 0.10C steel, (**d**) 0.16C steel, and (**e**) 0.3C steel. YD—the yield drop.

The stress–strain curves of the 0.05C steel, 0.10C steel, 0.16C steel, and 0.3C steel are shown in Figure 3a. To examine the yield point, the stress–strain curves around the yield plateau are enlarged in Figure 3b–e. The strain in Fig. 3 is the average value over the gauge length of 30 mm, and the stress–strain curves show the macroscopic response of the steels to the applied load. The enlarged stress–strain curves show that stress fluctuation is present on the yield plateau. A sharp yield-drop phenomenon occurred in the 0.05C steel, 0.10C steel, and 0.16C steel, but not in the 0.3C steel. 

The volume fraction of pearlite in the 0.05C steel is only 1.2%; thus, the effect of pearlite is weak, and it can be neglected. Among the four steels, the ferrite grain size decreased with an increase in carbon content. Previous studies [9,25] have shown that decreasing the grain size favors the occurrence of the yield-drop phenomenon. If the effect of grain size is strong enough, the 0.3C steel, whose ferrite grain size is the smallest, should have the yield-drop phenomenon. However, as shown in Figure 3e, an upper yield point is not present in the 0.3C steel. This indicates that, in addition to the effect of the grain size, there must be another factor whose effect is too strong to overcome the effect of grain size. The factor is the carbide (cementite). 

The lattice parameter of ferrite at room temperature is 2.866 Å, and those of cementite at room temperature are 4.5246, 5.0885, and 6.7423 Å [26]. The difference in the lattice parameters results in misfitting at the boundaries of ferrite and cementite, which induces internal stress. In Ref. 13, Mohsenzadeh et al. investigated the internal stress caused by the spheroidal cementite particles dispersed in the ferrite matrix as well as the internal stress-induced yielding (plastic deformation) of ferrite. Due to the plastic deformation of ferrite, a high density of unlocked dislocations forms around the cementite particles to maintain the lattice continuity, which favors the disappearance of the yield-point phenomenon. It was found that when there is a sufficient amount of cementite particles, the density of the increased mobile dislocation is high enough to eliminate the yield drop [13]. 

The cementite in the four steels used is mainly present in the form of a eutectoid structure (denoted as pearlite) composed of alternating layers of ferrite and cementite. The volume fraction of spheroidal cementite particles is very low, and the effect of spheroidal cementite particles can be neglected. The steels used in the present study consist of polycrystalline ferrite grains and pearlite colonies. Under an applied stress, there is mesoscopic strain incompatibility between ferrite and pearlite, and huge internal stresses are generated in pearlite all along the deformation [27]. In pearlite, there are numerous ferrite/cementite interfaces, and the interfaces play multiple roles in the deformation process, providing sites for the nucleation, absorption, and annihilation of dislocation. Atomistic simulation [28,29] and molecular dynamics simulation [30] have been used to characterize the interfaces. These studies indicate that the ferrite/cementite interfaces within pearlite can act as sites for dislocation emission. 

Microstructures with a higher fraction of pearlite may facilitate the easier generation of dislocation through a greater volume of ferrite/cementite interfaces, thus decreasing the degree of yield drop. Pearlite contributes to the elimination of the upper yield point in 0.3C steel. Note that the tensile tests were performed at quasi-static rates in the present study. At dynamic rates, the research carried out by Euser et al. [31] showed that pearlite has a similar effect on discontinuous yielding. 

### 3.3. Plastic Band

**Figure 4:**
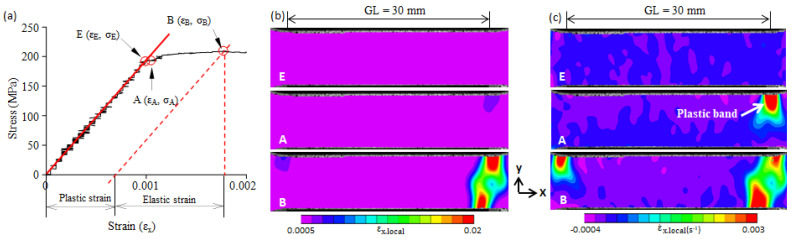
(**a**) The elastic limit point (E-point), the beginning point of plastic band formation (A-point), and the yield point (B-point) on the stress–strain curve of 0.05C steel; (**b**) the two-dimensional strain field at E-, A-, and B-point; (**c**) the two-dimensional strain-rate field at E-, A-, and B-point. GL—the gauge length of the extensometer used.

The stress–strain curve of the 0.05C steel from the zero point to the upper yield point is shown in Figure 4a. Stress linearly increases with an increase in strain from the zero point to the E-point. The E-point is the elastic limit point, under which the steel is in a macroscopically elastic state. From that point, stress deviates from the initial straight line to the upper yield point (B-point). This indicates that plastic deformation begins to occur and gradually increases from the E-point to the B-point. However, the stress–strain curve does not give detailed information regarding the plastic strain, such as, for example, the site of the local plastic concentration and the plastic strain distribution. 

The deformation process of the front surface of the tensile specimens was recorded with a camera. The obtained digital images were processed with two-dimensional DIC to calculate the local strain field and the local strain-rate field of the front surface of the tensile specimens. Three points (E, A, and B) on the stress–strain curve in Figure 4a were selected. The fields of the local strain ($\varepsilon_{x.\mathrm{local}}$) and the local strain-rate (${\dot{\varepsilon }}_{x.\mathrm{local}}$) along the tension direction (the x-axis) at the four points are shown in Figure 4b,c, respectively. The range of the gauge length (GL) of the extensometer used is given in Figure 4b,c. 

**Figure 5:**
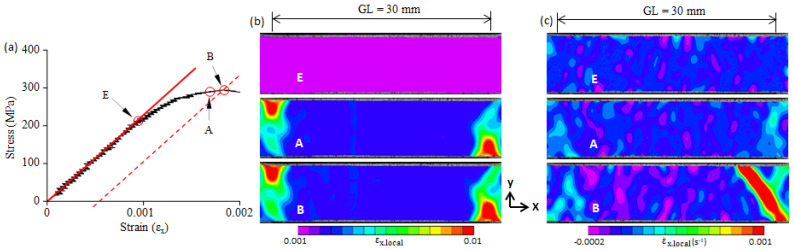
(**a**) The elastic limit point (E-point), the beginning point of plastic band formation (A-point), and the yield point (B-point) on the stress–strain curve of 0.10C steel; (**b**) the two-dimensional strain field at the E-, A-, and B-point; (**c**) the two-dimensional strain-rate field at the E-, A-, and B-points. GL—the gauge length of the extensometer used.

**Figure 6:**
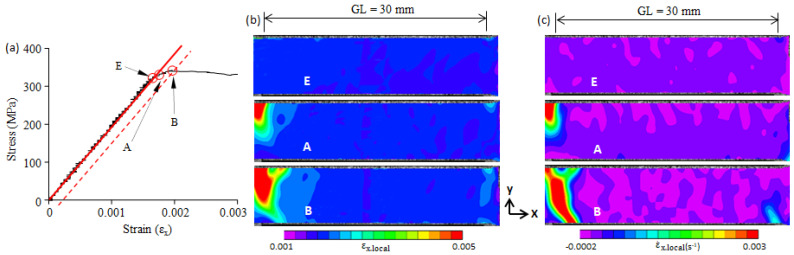
(**a**) The elastic limit point (E-point), the beginning point of plastic band formation (A-point), and the yield point (B-point) on the stress–strain curve of 0.16C steel; (**b**) the two-dimensional strain field at the E-, A-, and B-points; (**c**) the two-dimensional strain-rate field at the E-, A-, and B-points. GL—the gauge length of the extensometer used.

**Figure 7:**
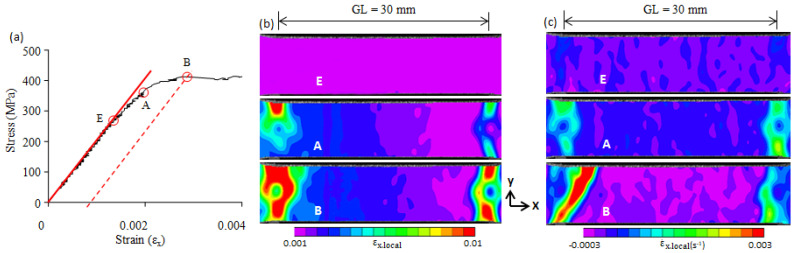
(**a**) The elastic limit point (E-point), the beginning point of plastic band formation (A-point), and the yield point (B-point) on the stress–strain curve of 0.3C steel; (**b**) the two-dimensional strain field at the E-, A-, and B-points; (**c**) the two-dimensional strain-rate field at the E-, A-, and B-points. GL—the gauge length of the extensometer used.

A two-dimensional local strain field gives the strain distribution on the x-y plane, but it cannot identify a moving plastic band. It has been verified that a two-dimensional local strain-rate field can identify moving plastic bands [22]; thus, we used a strain-rate field (Figure 4c) to identify the formation and propagation of a plastic band. The E-point is the elastic limit point. The local strain ($\varepsilon_{x.\mathrm{local}}$) and local strain-rate (${\dot{\varepsilon }}_{x.\mathrm{local}}$) fields at the E-point show that the $\varepsilon_{x.\mathrm{local}}$ and ${\dot{\varepsilon }}_{x.\mathrm{local}}$ were nearly uniform, and no plastic band was formed. As the applied stress continued to increase, plastic deformation began to take place in the right-upper corner and gradually increased, but no plastic band was nucleated. When the applied stress reached the A-point, a plastic band (Lüders band) was formed at a site with a plastic strain concentration (upper-right corner) (see A-point in Figure 4b,c). The A-point is the starting point of the Lüders band formation. From the A-point to the B-point, plastic strain concentration continued, and the Lüders band propagated across the specimen width. Similar deformation behaviors were found in the 0.10C steel, 0.16C steel, and 0.3C steel, and they are shown in Figure 5, Figure 6 and Figure 7. Note that because there is no upper yield point in the 0.3C steel, the B-point in Figure 7 is the first peak on the stress–strain curve (cf. Figure 3e). 

**Figure 8:**
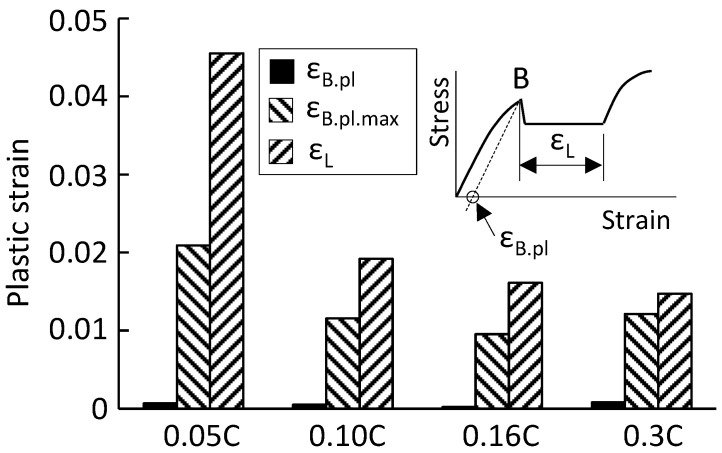
The Lüders strain and the plastic strain at the yield point (B-point) of 0.05C steel, 0.10C steel, 0.16C steel, and 0.3C steel. ε_B.pl_—the average plastic strain over the gauge length at the B-point obtained from the stress–strain curve; ε_B.pl.max_—the maximum local plastic strain at the B-point; ε_L_—the Lüders strain.

As shown in Figure 4, Figure 5, Figure 6 and Figure 7, a great concentration of plastic strain is present at the upper yield point. The plastic strain at the B-point is evaluated in terms of the average plastic strain (ε_B.pl_) over the gauge length and the maximum local plastic strain (ε_B.pl.max_) in the present study. The ε_B.pl_ was obtained from the stress–strain curve as shown in Figure 4a, and the ε_B.pl.max_ was determined from the two-dimensional local strain field. The values of ε_B.pl_ and ε_B.pl.max_ of the four steels used are plotted in Figure 8. A previous study showed that the maximum local strain in a plastic band located on the yield plateau can reach the Lüders strain [19]. For comparison, the Lüders strain (ε_L_) is also given in Figure 8. It shows that the maximum local plastic strain (ε_B.pl.max_) is vastly greater than the average plastic strain (ε_B.pl_), but it is smaller than the Lüders strain (ε_L_). 

**Figure 9:**
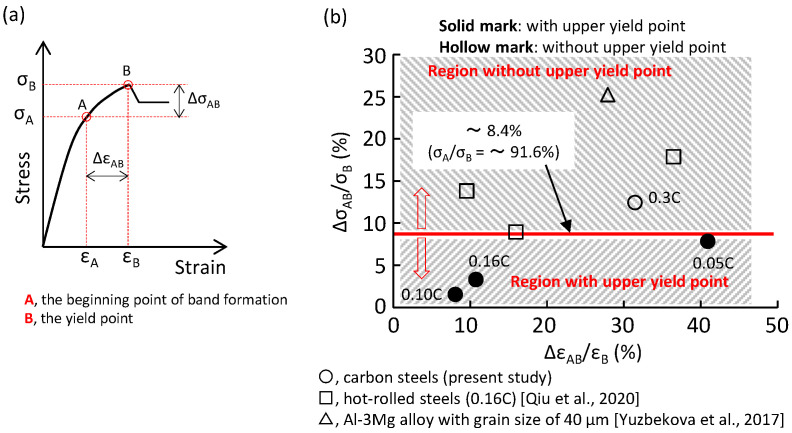
(**a**) Illustration of the beginning point of plastic band formation (A) and the yield point (B) on the stress–strain curve, and (**b**) the relationship between the yield-point phenomenon and the beginning point of plastic band formation.

Figure 4, Figure 5, Figure 6 and Figure 7 show that a plastic band formed ahead of the yield point irrespective of the presence of the upper yield point. Therefore, we cannot say that the early formation of the plastic band inevitably eliminates the upper yield point. In Figure 9a, the deviation of the A-point from the B-point is represented in terms of Δε_AB_/ε_B_ and Δσ_AB_/σ_B_. The Δε_AB_/ε_B_ and Δσ_AB_/σ_B_ of the four steels used (○ mark) are plotted in Figure 9b. The solid marks and hollow marks represent if the material does or does not have an upper yield point, respectively. The experimental data in the literature are also given in Figure 9b. Apparently, there is a critical diving line (σ_A_/σ_B_), above (or below) which the upper yield point is absent (or present). The critical stress ratio of σ_A_/σ_B_ is about 0.92. The upper yield point is the result of an abrupt increase in the dislocation density. If the increase in the dislocation density is a slow process, an abrupt increase cannot be induced; as a result, the upper yield point will be absent. When a plastic band occurs at a low stress level, plastic deformation from this point to the yield point is a slow process, which does not favor the appearance of an upper yield point. 

## 4. Conclusions

The yield-point phenomenon and the local deformation behavior around the yield point in four ferrite–pearlite steels with different carbon contents were investigated by means of stress–strain curves and the local strain and local strain-rate fields. The results are summarized as follows:

1. The degree of the yield drop decreased with an increase in the pearlite fraction. When the pearlite fraction was sufficiently large, the upper yield point disappeared.
2. Large local plastic strain was present at the yield point. It was much larger than the average plastic strain over the gauge length, and it was smaller than the Lüders strain.
3. The yield-point phenomenon was related to the formation of a plastic band. A plastic band is formed at a certain stress level (i.e., the critical initiation stress) that is smaller than the upper yield stress (σ_B_). When the critical initiation stress was larger than 0.92σ_B_, the upper yield point disappeared.

## Figures and Tables

**Table 1:** Chemical composition and heat-treatment conditions of the steels used in the present study.

	Chemical Composition (in wt%)			Heat-Treatment Procedure
	C	Mn	Si	
0.05C steel	0.05	0.008	0.002	900 °C, 5 min → furnace cooling to room temperature
0.10C steel	0.10	1.53	0.44	900 °C, 5 min → furnace cooling to room temperature
0.16C steel	0.16	1.46	0.44	900 °C, 5 min → furnace cooling to room temperature
0.3C steel	0.3	1.5	0.3	850 °C, 5 min → furnace cooling to room temperature

The size of the samples used for heat treatment is 120 mm(L) × 50 mm (W) × 12 mm (t).

**Table 2:** Ferrite grain size and volume fraction of cementite, ferrite, and pearlite.

	D_PF_ (μm)	f_ce_ (%) ^*^	f_PF_/f_P_
0.05C steel	30	0.8	98.8/1.2
0.10C steel	18	1.5	92/8
0.16C steel	9	2.4	83/17
0.3C steel	4	4.6	68/32

D_PF_—average grain size of polycrystalline ferrite grains; f_ce_—volume fraction of cementite; f_PF_—volume fraction of polycrystalline ferrite grains; f_P_—volume fraction of pearlite; ^*^, f_ce_—calculated by 1.946C/(12.72 + 0.046C), C in wt% [23].

## Data Availability

Data sharing is not applicable to this article.
